# Medical and Physical Intelligence

**Published:** 1802-05

**Authors:** 


### MEDICAL AND PHYSICAL INTELLIGENCE.

### [ FOREIGN AND DOMESTIC. ]

### A NEW PREPARATION OF BARK.

Meſſrs. REECE and DURIE, of Henrietta Street, Covent Garden, have communicated to the Editors a new preparation of the Peruvian Bark, termed by the French chemiſts, Sel de la Garraye, or Sel Eſſentiel de Cinchone; the former from its firſt having been made by the Count de la Garraye, and the latter from its containing the active or eſſential parts of the bark, and its deliqueſcent property on expoſure to the atmoſphere.—This volatile extract is obtained from a cold infuſion of the bark carefully evaporated to a ſtate of dryneſs by ſo gentle a heat that its volatile particles may not be diſſipated or injured; and in order to render its expoſure even to this gentle heat as ſhort as poſſible, the evaporation is directed to be performed on a wide ſurface, as a large earthen plate; and when reduced to a perfect ſtate of dryneſs, it ſhould not be thicker than thin glaſs, and nearly as tranſparent.—The produce of a pound of the beſt pale bark, not exceeding more than 9 drachms, muſt of courſe enhance its value; but 10 grains being equivalent to a drachm of the powder, it will not prove in practice much more expenſive.

The Peruvian Bark as a tonic medicine is more extenſively and ſucceſsfully employed than any other article in the Materia Medica, but through the bulk of a full doſe of the powder mechanically irritating the fauces, ſtomach, and inteſtines, too often produces nauſea, vomiting, or diarrhœa, ſo much to be dreaded in caſes of extreme debility of the ſyſtem as Typhus Fever, &c. In ſuch caſes no other preparation of this valuable medicine affords a proper ſubſtitute. Through the great heat employed by chemiſts (for the ſake of diſpatch) in the preparation of the common extract, its volatile latile parts are diſſipated, on the preſervation of which its activity depends. The tincture, on account of the quantity of ſpirits retaining a proper doſe of the extractive matter, is in many caſes inadmiſſible. The decoction, by depoſiting its reſin on cooling, and if haſtily made, (too often the caſe by the apothecary's apprentice, or the apprentice's boy) will prove little better than aqua *pura*, while a doſe of the cold infuſion, adequate to 10 grains of the volatile extract, would be more than the ſtomach would retain, or the patient perſuaded to ſwallow.—From theſe great objections this volatile extract is certainly exempt; and after the powder has been rejected or proved cathartic, it may with equal advantage be preſcribed.—It is eaſily ſoluble in either a vinous or aqueous menſtruum; in port wine it forms a grateful tincture; in camphorated julep, an efficacious mixture in ſynochus and typhus fevers; and in ſimple cinnamon water, a pleaſant tonic medicine.—In two caſes of phthiſis pulmonalis, it has been adminiſtered with ſucceſs in checking the colliquative perſpiration and diarrhœa, without aggravating the cough, as the following mixture:

℞. Ext. Cinchonæ volat. ʒj. Tinct. digitalis ʒji. Pulv. g. Arabic. Syrup. papav. alb. aa. ℥ſs. Aquæ anethi ℥jv. M. cap. cochl. ij. larg. ter die.

They have lately prepared a quantity of this volatile extract, which may be examined by any profeſſional gentleman.

They have the ſame preparation of the yellow and red bark, the caſcarilla and anguſtura, in forwardneſs. They have likewiſe juſt expreſſed a quantity of the *oleum e seminibus ricini* without heat, which is free from the unpleaſant acrid taſte and dark colour of the caſtor oil imported from the Weſt Indies.

---

### Composition of Urine.

Mr. Proust, of Madrid, has made ſome intereſting experiments concerning the conſtituent particles of urine, which he found to be the following:

1. *Sulphur.*—The urine contains this ſubſtance, moſt probably generated in the body by the action of the vis vitalis, in a ſimilar manner as phoſphorus, iron, and carbonic acid, which we exhale in perſpiration. Freſh urine blackens ſilver veſſels; and when boiled in them ſmall particles of ſulphurated ſilver are diſengaged. When the urine has been ſtanding for about a fortnight it effervesces with acids, and the ſulphur goes off with carbonic acid; and on applying over the orifice of the veſſel, a ſheet of paper, which has been written on with a ſolution of lead, the traces of ſulphur will be immediately perceived. In order to be quite ſure of the colour communicated to the lead having aroſe from ſulphur, and not from the oily vapours which are diſengaged during the fermentation, Mr. P. wrote, on the ſame ſheet, with a ſolution of antimony and tin, but the letters became yellow and brown, in the ſame manner as as when the oxyds of thofe metals are expofed to fulphurous vapours.

2. *Carbonic acid* is obferved in great abundance in the urine, from which it feparates on its being difcharged. It is owing to the prefence of carbonic acid, that on evaporating the urine, an ebullition is perceived, fometimes fo violent as to make it boil over.

3. *Ammonia.*—When urine is expofed in fummer to the action of the atmofphere, for about a fortnight, the ammonia increafes confiderably, and a few drops of fulphuric acid occafion an ebullition in it, and carbonic acid with fulphur is difengaged; but when the veffel is fhut againft the contact of air, the urine never undergoes any change in all feafons, as the atmofphere contributes to the generation of ammonia by means of the azote with which it is impregnated.

4. *Carbonat of Lime.* — When urine is preferved in new veffels, cryftals begin to adhere to their fides; they are of a prifmatic form, and fall to pieces in open air; they confift of carbonat of lime, a fubftance which is alfo difcovered in calculous concretions and in bones. Mr. P, however, could never find any fulphat of natron in the urine.

5. *A rofe-coloured fubftance.*—Thus Mr. P. calls the fubftance, which is depofed in the urine during feveral febrile ftates, after it has become cold and known under the name of lateritious fediment. In a found ftate of the body this fediment is not frequently obferved, partly on account of its great folubility in water, and partly on account of its affinity to the ammonia, by which it is kept diffolved in cold urine; but in a febrile ftate, where it is more abundantly generated, it eafily feparates, and is found to adhere to the fides of the veffel: perhaps the urine is not fufficiently provided with ammonia to keep it diffolved. On heating the urine the fediment diffolves again; but when it falls to the bottom of the veffel it is always mixed with uric acid and phofphat of lime. Alkalis may be faturated with the rofe-coloured fubftance, but never produce cryftallifations with it; from this combination, however, it is feparated by acids, exhibiting a yellowifh powder. On pouring a few drops of nitric acid to frefh urine, a precipitation enfues, confifting of nitric acid and the rofe-coloured fubftance, while the phofphat of lime remains diffolved in the liquor, from which it may be precipitated by alkalis. It is probable, that as the lithic acid and the rofe-coloured fubftance are at the fame time feparated from the urine, they are diffolved by ammonia or any other alkali, in a fimilar way as the phofphoric acid keeps the lime diffolved as phofphat of lime. The lithic or uric acid being lefs foluble in water than the rofe-coloured fubftance, may be eafily feparated by pouring hot water on the fediment, and wafhing it feveral times on the filtre, and that acid will remain; it is befides eafily diftinguifhed by its grey colour, cryftalline appearance, and the horny fmell which it yields on being moiftened. The rofe-coloured fubftance could could never be traced in urinary calculi, at leaſt thoſe that were examined by Mr. Prouſt.

6. *Acetous acid* is diſcovered in the urine, but in very little quantity, when it is inſpiſſated; or by mixing one part of freſh urine with a little ſulphuric acid and diſtilling the mixture; the water that goes over at firſt has a particular ſmell, to which ſucceeds a ſouriſh liquor, ſmelling like vinegar. In order to obtain this acid in a greater quantity, drop, by degrees, concentrated ſulphuric acid into freſh urine which has been previouſly inſpiſſated, and after having freed it from the ſalt cuticula, dilute it to the conſiſtency of a thin ſyrup; this mixture being expoſed for ſome days to open air, exhales a very characteriſtic odour of vinegar, becomes of a lighter colour, and depoſits cryſtals o i the ſides of the veſſel, and on the bottom a reſinous ſubſtance. On diſtilling the liquor by a gentle fire, after a previous ſeparation from the ſediment, a conſiderable portion of this curious vinegar is obtained, of which the reſiduum yields ſtill a little, by repeated diſtillation. In order to aſcertain the identity of this liquor with vinegar, the following experiment was thought deciſive. A portion of this acid was ſaturated with carbonat of copper, which gave a green ſolution, and on being diſtilled, a conſiderable quantity of grainy, not liquefiable, concretions of a green colour were obtained; which, though different from true acetat of copper, yielded by means of ſulphuric acid, and by diſtillation, an acid as penetrating and agreeable as vinegar, ſo that we have every reaſon to think its elementary particles the ſame with thoſe of acetous acid.

7. *The resin which colours the urine.*—During the diſtillation of the abovementioned acid, a kind of reſin ſeparates, reſembling extremely, with reſpect to conſiſtency and ſmell, the reſin of caſtoreum. It is eaſily ſoluble in alkohol, from which it is ſeparated by water, in which, however, it ſhows a degree of ſolubility, like the reſin of gall, which makes us conclude that it is the ſame with it, and that its particular ſmell and colour is owing to the admixture of other parts of the urine, and it is, in all probability, the ſubſtance from which the colour of the urine originates, and ſeems to be the ſame with the urée, lately diſcovered by Fourcroy and Vauquelin.

---

### Acetic Acid.

The following new and ſimple method of preparing radical vinegar, or acetic acid has lately been publiſhed in the *Annales des Arts et Manufactures*:—Take any quantity of white vinegar, concentrated by the froſt, and pour to it half as much concentrated ſulphuric acid; then diſtil the mixture in a ſand bath till the vapours of the ſulphurous acid began to appear, when a light and ſtrong ſcented liquid is obtained, which, however, requires to undergo a ſecond diſtillation before it is the real acetic acid. It has not yet been aſcertained, whether the expence incurred be greater or leſs by this, than by the common method of obtaining radical vinegar, in which the acetite of copper is uſed. But it is certain, that acetic acid obtained by the new method may be uſed without the apprehenſions henfions excited by that fold commonly in the fhops. The French chemift warns the ladies, who ufe it as a luxury, to reflect, that, when refpiring its odour, they introduce into their lungs more or lefs copper, one of the moft powerful poifons; at the fame time he urges them to difcountenance the former procefs, in order to introduce the acid made by himfelf.

---

*New Metal.*—Mr. HATCHET has difcovered a new metal, or at leaft an acidifiable oxyd, in the analyfis of a mineral from North America. It promifes to be a ufeful difcovery, as it affords orange and green coloured precipitates of great beauty and permanence. Mr. Hatchet propofes to call it Columbium. In external appearance this mineral refembles chromate of iron.

---

*Prefervation of water.*—The Society for the Encouragement of Arts, &c. has awarded a gold medal to Gen. Bentham for a new method of preferving water perfectly fweet during long voyages. The experiment was tried on board two floops of war, the Arrow and the Dart, and appears to have terminated in the moft fatisfactory manner. Inftead of the ordinary ftowage in cafks, fixteen tanks or cafes, adapted to the fhape of the hold, were placed in each veffel, and filled with about forty tons of water, by means of which the water occupied much lefs room in the fhips than it would have done if cafks had been made ufe of. The tanks were made of wood, accurately lined with fheets of tinned copper, all the junctures of which were fecured by folder, fo that the water was no where in contact with any thing but the furface of tin. By way of comparifon, about 30 tons of water were ftowed on board each veffel, in cafks as ufual. The water in all the tanks on board one fhip, and that in thirteen of the tanks on board the other, was uniformly found to continue as pure as when it was firft taken from the fpring: that which was contained in the other three tanks was more or lefs tainted, as that in the cafks was. After the water had remained on board a fufficient length of time, it was ufed out, and the tanks replenifhed from time to time; but in fome of the tanks, the water was allowed to continue three years and a half; 25 gallons of which, being fent to the Society, was found to be ftill wholly unaltered.

---

*Mode of cleanfing water.*—The water in Paris, and many parts of France, is intolerably turbid and foul; the following is a method adopted to filter it in large quantities:—In conftructing a well of five feet diameter, the excavation ought to be from 12 to 15 feet. A falfe well is made, 10 or 12 feet in diameter; in the middle of this the real well is conftructed in fuch a manner, that the water may filter through the interftices left between the ftones which form the outfide of the inner well; the falfe well is then filled with fand and pebbles, fo that the water muft firft filter through them before it reaches the real well. This method has been found to produce great plenty of water, perfectly clear and free from all extraneous matters.

*Electricity.*

*Electricity.*—From a ſtatement of the experiments made by the Rev. Mr. BENNET on the electricity produced by the contact of metals previouſly to the year 1789, and alſo of thoſe made by Mr. CAVALLO previouſly to 1795, Mr. NICHOLSON draws the following concluſions:—1. That the contact of one metallic ſubſtance with another generally produces electricity; 2. That the quantity and quality of the electricity ſo produced are various, according to many circumſtances which ſeem to occur in the product of it, or in a great meaſure to influence it; 3. And that theſe circumſtances are the various nature of the metallic ſubſtances, their various degrees of heat, the ſtate of the atmoſphere, the hand of the operator, &c. each of which cauſe has its ſhare in the reſult.

---

*Suppoſed Diſcovery of the Mode of rendering harmleſs the Poiſon of Serpents.*—It is well known, that in Egypt, India, and the hotter parts of America that abound with poiſonous ſerpents, there are certain individuals, who poſſeſs the power of entirely diſarming theſe formidable animals, and are able to handle them with perfect impunity at the very time that any other perſon, approaching them incautiouſly, would be fatally convinced of their ability to deſtroy. This happy exemption is attributed by the people themſelves to the preſervative effects of certain vegetables, the knowledge of which has hitherto been carefully concealed. Many of the European philoſophers have, however, treated the affair as a mere juggle. This ſtate of uncertainty is now, happily for humanity and ſcience, relieved by the moſt important communication from Don Pedro d'Orbies y Vangas, through the medium of Count Rumford, which, if entirely to be depended upon, will entitle the communicator to rank high among the benefactors of mankind. Don Pedro is a native of Santa-Fe, and, in the year 1788, being at Margarita, he met with a ſlave who poſſeſſed the power of charming the moſt venomous of the American ſerpents; after the Negro had exhibited his ſkill, he was induced by a reward to promiſe to diſcover his ſecret. The next morning he returned with the leaves of a plant, called *vejuco du guaco*, and having bruiſed them, in the preſence of Don Pedro, gave him two large ſpoonfuls of the juice to drink; then making three inciſions between the fingers of each hand, he inoculated the Spaniard with the ſame juice, and performed a ſimilar operation on each foot, and on each ſide of the breaſt, after which he informed him that he was no longer acceſſible to the poiſon of ſerpents. Don Pedro then, after making the Negro anſwerable for any ill conſequences, took into his hands ſeveral times one of the ſerpents that had been brought by the ſlave the day before, without receivtng the ſmalleſt injury from the animal. Encouraged by this firſt attempt, two domeſtics, being in like manner prepared by the guaco-juice, went into the fields, and ſoon returned with another kind of ſerpent, equally venomous with the former, without ſuſtaining any hurt; another perſon, being ſimilarly prepared, and afterwards bitten by by a poifonous ferpent, received no further injury than a flight local inflammation. Since this period, Don Pedro has repeatedly caught ferpents with his own hands with abfolute impunity, employing no further preparation than merely drinking a little of the guaco-juice. The plant, whofe effects are thus attefted, has not as yet been admitted into any botanical fyftem, but is amply defcribed in a memoir by the Spanifh gentleman already mentioned, inferted in a weekly paper publifhed at Santa-Fe. It is of the compound-flowered or fyngenefious clafs. The ftamina are five in number, united by their anthers into a cylinder, through which rifes the piftill with a deeply divided fummit. The corolla is monopetalous, infundibuliform, with five indentations, and of a yellow colour; each calix contains four florets, and feveral of thefe grow together, forming a corymbus: the feeds are broad and feathered; the root is fibrous, perennial; the ftem ftraight, cylindrical when young, but, when old, becomes pentagonal: leaves are heart fhaped, oppofite, of a dark-green mixed with violet, velvetty on the upper furface. It grows by the fides of rivulets, and in fhady places, in the viceroyalty of Santa-Fe.

---

*Culture of Exotics in Corfica.*—It is about a year ago fince the French government caufed to be tranfported to Corfica a collection of exotic vegetables, which were furnifhed by the Mufeum of Paris, and felected from fuch as appeared the moft likely to become inured to the climate of that ifland, and to be, at the fame time, the moft ufeful to the arts and to the commerce of the inhabitants. Citizen Noifette, gardener, was appointed to attend them, and to fuperintend their culture. In a letter addreffed to Citizen Thouin, and dated Ajaccio, the 10th of Brumaire laft, he gives a detail of his firft fucceffes. Almoft all thofe vegetables are turned to good account; they were planted immediately in a platform or mount, and this tranfplantation does not appear to have diminifhed their vigour; on the contrary, their growth has been fenfibly obferved during the firft year. Among the trees which compofe this vegetable colony, we diftinguifh the fweet-acorned-oak, the falfe accaia, the cytifus of the Alps, the jujube-tree (*le jujubier*) the Judæa-tree (*arbre de Judée*) the *goyavier*, the indigo-plant (*l'indigotier*) the cotton fhrub, the fophora of Japan, the *plaque minier*, of Virginia, and the bean-plant of China (*fevier de la China.*) Among the plants we reckon the nopal of cochineal, the pit-aloes, different fpecies of *arum*, of *asclepias*, of *geranium*, of *folanum*, and of *belladona;* fome of which are ufeful in the arts, and others in medicine. The multiplication and naturalization of thefe vegetables, all foreign to Corfica, will, doubtlefs, call for much care and conftancy; but every thing may be expected from the experience of the gardener to whom they are entrufted, efpecially if he fhall obtain from the adminiftrators of that ifland, as there is no reafon to doubt, the neceffary affiftance to accomplifh this defign.

---

*Improved*

*Improved Edition of Lagrange's Chemiſtry.*—There has lately appeared at Paris the ſecond edition, conſiderably augmented, of A Manual of a Courſe of Chemiſtry; or, the Elementary Principles, in Theory and Practice, of that Science, by Citizen BOUILLON LAGRANGE. This new work, however, muſt not be confounded with that which appeared about two years ago, under the ſame title, as it differs eſſentially from it, both in the plan which the author has adopted, and in the manner in which he has executed it. In his firſt Manual, Citizen Bouillon Lagrange only intended to preſent exact deſcriptions of all the proceſſes, by means of which ſuch other reſults were to be obtained. The avidity with which that work was brought up, quickly demonſtrated the public conviction of its utility. The firſt edition being now entirely out of print, the author has judged it neceſſary to publiſh a ſecond; but wiſhing to render it more intereſting, he has made it his buſineſs to add ſome illuſtrations, which appeared to him proper to favour the ſtudy of chemiſtry, and to accelerate the progreſs of that ſcience.

---

Mr. HEY, ſenior-ſurgeon to the General Infirmary at Leeds, has now in the preſs a volume of Obſervations in the Practice of Surgery, illuſtrated by caſes.

---

Dr. JOHN MURRAY, Lecturer on Natural Philoſophy and Chemiſtry at Edinburgh, has made a great number of experiments to aſcertain whether fluids be or be not conductors of caloric. The experiments which he has laid before the public are very curious, and ſeemingly made with great accuracy, and are ſufficient to controvert the opinion advanced by Count Rumford, that fluids are non-conductors of caloric. We may in a future communication, expect the detail of a ſeries of concluſive experiments made to determine the point.

---

Dr. MARSHALL, who for the two laſt years has been extending the Vaccine Inoculation through the Britiſh poſſeſſions in the Mediterranean and through the ſouthern part of Europe, has lately taken up his reſidence at Paris.

